# Repurposing Tecfidera for cancer

**DOI:** 10.18632/aging.101001

**Published:** 2016-07-16

**Authors:** Laurence Booth, Mark Malkin, Paul Dent

**Affiliations:** Departments of Biochemistry and Molecular Biology, Virginia Commonwealth University School of Medicine, and the Massey Cancer Center, Richmond, VA 23298, USA

**Keywords:** dimethyl fumarate (DMF), monomethyl fumarate (MMF), Tecfidera, ruxolitinib, Jakafi, glioblastoma multiforme, oncology

The compound dimethyl fumarate (DMF) has been an approved drug in Europe for several decades as a therapeutic for psoriasis and relapsing-remitting multiple sclerosis (MS). More recently, the drug has been approved in The United States as an MS treatment (Tecfidera®). Upon ingestion, the drug is rapidly metabolized into the biologically active form of the agent, mono methyl fumarate (MMF); the biological actions of DMF and MMF in vitro are not identical, for example DMF modulates NFκB activity to a much greater extent than MMF. We have published two studies in the last two years describing how MMF can be re-purposed from its use as an MS therapy into an anti-cancer drug [1, 2]. It is noteworthy that another recently approved anti-MS drug, Fingolimod (Gilenya®), that acts through down-regulation of sphingosine-1-phosphate signaling, also has anti-cancer properties and can synergistically combine with MMF to kill many tumor types, including multiple genetically diverse primary human glioblastoma cell isolates. As a single agent MMF was also able to kill activated microglia freshly isolated from patient tumors which was paralleled by the drug initially reducing the expression of IL-6, TNFα and TGFβ. At present a group at Johns Hopkins Hospital is performing a phase I clinical trial combining Fingolimod with the standard of care Stupp protocol in newly diagnosed glioblastoma patients (NCT02490930).

High concentrations of DMF are known to profoundly modulate cellular Redox signaling with regulation of Nrf2 playing a key role in its anti-cancer properties. However, the precise mechanisms by which clinically relevant concentrations of the biologically active form of the drug MMF act as an anti-cancer agent are still not fully understood. We discovered as a single agent MMF caused modest changes in the activities of well-recognized signal transduction pathways, with ERK1/2 and AKT being transiently inactivated and the expression of BCL-XL reduced. However, when combined with the JAK1/2 inhibitor ruxolitinib (Jakafi®), a drug used to treat myeloproliferative disorders, the inhibition of ERK1/2 and AKT signaling was prolonged, as was the inactivation of STAT3 and STAT5. Inactivation of these cyto-protective signaling modules directly caused an increase in the expression of pro-cell death BIM, an activation of pro-cell death BAD and decreased expression of the cyto-protective proteins superoxide dismutase 2, thioredoxin, BCL-XL and MCL-1. These changes in biology resulted in very high levels of toxic reactive oxygen being produced, the activation of pro-cell death BAX, and ultimately tumor cell execution that utilized both AIF and caspase 3 downstream of the mitochondrion.

In our earliest work with MMF we found the drug could synergistically kill tumor cells by interacting with proteasome inhibitors, the NSAID celecoxib (Celebrex®) as well as with the standard of care brain tumor therapeutics temozolomide (Temodar®) and ionizing radiation. When combined with proteasome inhibitors, MMF again was shown to inactivate ERK1/2, AKT, STAT3 as well as mTOR, which resulted in increased levels of toxic autophagosome production. Unlike its combination with ruxolitinib, MMF combined with proteasome inhibitors caused activation of the death receptor CD95 which signaled through the mitochondria to cause AIF-dependent, but caspase 3-independent, tumor cell execution. As a result of these initial studies a phase I trial is open at Massey Cancer Center in which newly diagnosed glioblastoma patients receive DMF together with the standard of care Stupp protocol, and is presently recruiting patients (NCT02337426). There are other ongoing clinical studies elsewhere using the drug as an anti-cancer agent for refractory leukemia/lymphoma (NCT02784834).

Thus the potential for use of DMF as an anti-cancer agent at first glance appears to be bright. Based upon our most recent publication, we believe that our next logical step is to propose a new 3 + 3 phase I trial in glioblastoma patients in which increasing doses of ruxolitinib are added to the RP2D of the [DMF + Stupp] protocol. However, whether InCyte and Biogen, the owners of Jakafi and Tecfidera, will provide drugs necessary for such a study is presently unknown. Novartis, the owners of Gilenya, have been reluctant in the past to provide their drug for any cancer-related clinical studies, probably due to its cardiac black box warning, and thus the likelihood that any trial combining Fingolimod with the RP2D of [DMF + Stupp] is remote. Possibly a more feasible next step to advance DMF use against glioblastoma, based on cost, may be to perform a new trial combining celecoxib with the RP2D of [DMF + Stupp]. We have recently shown that high, but still clinically relevant, concentrations of celecoxib (2-5 μM) can enhance the anti-cancer properties of MMF as well as the combination of [sorafenib (Nexavar®) + sildenafil (Viagra®)] [3]. At present we have an open phase II trial combining [sorafenib + sildenafil + valproate (Depakote®)] in recurrent malignant glioma (NCT01817751) and [regorafenib (Stivarga®) +sildenafil] in all solid tumors (NCT02466802) [4–7].

In conclusion, we have shown that the MS approved drug DMF can be re-purposed as an anti-cancer agent. DMF is not the only non-cancer drug we have recently repurposed and translated to the clinic. Ruxolitinib, celecoxib, valproic acid and sildenafil are medications for myeloproliferative disorders, arthritis, bipolar disease and erectile dysfunction, respectively, and all in a rational manner can be combined with each other or with established cancer therapies to kill tumor cells. It is hoped that our initial studies in the area of repurposing, combined with those of other groups, may yield better and novel ways to treat cancer.
